# Calciotropic and Phosphaturic Hormones in End-Stage Heart Failure Patients Supported by a Left-Ventricular Assist Device

**DOI:** 10.1371/journal.pone.0164459

**Published:** 2016-10-27

**Authors:** Armin Zittermann, Jana B. Ernst, Stefan Pilz, Jens Dreier, Joachim Kuhn, Cornelius Knabbe, Jan F. Gummert, Michiel Morshuis, Hendrik Milting

**Affiliations:** 1 Clinic for Thoracic and Cardiovascular Surgery, Heart- and Diabetes Center NRW, Ruhr University Bochum, Bad Oeynhausen, Germany; 2 Division of Endocrinology and Diabetology, Department of Internal Medicine, Medical University of Graz, Graz, Austria; 3 Institute for Laboratory and Transfusion Medicine, Heart- and Diabetes Center NRW, Ruhr University Bochum, Bad Oeynhausen, Germany; 4 Erich and Hanna Klessmann Institute for Cardiovascular Research and Development, Heart- and Diabetes Center NRW, Ruhr University Bochum, Bad Oeynhausen, Germany; Boston University, UNITED STATES

## Abstract

**Background:**

Calcium and phosphate are central for myocardial contractility and energy metabolism, and low levels of the calciotropic hormone 1,25-dihydroxyvitamin D (1,25(OH)_2_D), as well as high levels of the phosphaturic hormone fibroblast growth factor (FGF)-23, are independently associated with poor clinical outcome in heart failure (HF) patients. We therefore aimed to investigate the postoperative time course of the aforementioned hormones in HF patients supported with a left-ventricular assist device (LVAD) implant.

**Methods:**

For the present study, stored biobank plasma samples of 69 patients, collected before LVAD implantation (t0) and 12 days (t1), 30 days (t2), 83 days (t3), and 300 days (t4) post-intervention, were used to measure circulating FGF-23, parathyroid hormone (PTH), 25-hydroxyvitamin D (25OHD), 1,25(OH)_2_D, and kidney function.

**Results:**

Most patients were male and had baseline INTERMACS levels and cardiac index values ≤ 3 and ≤ 2.7 L/min/m^2^, respectively. There were significant time effects on estimated glomerular filtration rate (eGFR), FGF-23 and 1,25(OH)_2_D, but not on PTH or 25OHD. Notably, eGFR values increased and FGF-23 levels decreased only transiently, whereas 1,25(OH)_2_D increased continuously until t4. The rise in 1,25(OH)_2_D was largely influenced by those patients who survived the first post-implant year, and was not seen in non-survivors. Variations in 1,25(OH)_2_D levels could only partly be explained by eGFR values or FGF-23, 25OHD, and PTH levels (multiple R^2^ = 0.305;P<0.001).

**Conclusions:**

The present study indicates that LVAD implantation has only transient effects on circulating FGF-23 levels, but is associated with a continuous increase in circulating 1,25(OH)_2_D levels, especially in survivors.

## Introduction

Heart failure (HF) is a chronic condition in which the heart muscle is unable to pump effectively to meet the body's need for blood and oxygen. Disturbances in several metabolic systems occur in HF patients, among them alterations in calcium (Ca) and phosphate metabolism [1]. Briefly, in isolated myocytes from patients with end-stage HF systolic ionized Ca transients are markedly reduced, diastolic Ca levels are increased, and the rate of diastolic decay of Ca is slowed compared with heart cells from healthy subjects [2]. The sarcoplasmatic reticulum Ca leak is considered to be an important patho-mechanism in HF, and this leak is similar in human ischemic cardiomyopathy and dilated cardiomyopathy [3]. Moreover, cardiac high-energy phosphate metabolism is altered in HF [4] and elevated serum phosphate levels have been reported in these patients [1].

The active, hormonal form of vitamin D, 1,25-dihydroxyvitamin D (1,25(OH)_2_D), plays a pivotal role in cardiac function. Cardiac muscle cells possess a 1,25(OH)_2_D receptor and a 1,25(OH)_2_D-dependent Ca-binding-protein [5]. In addition, a 1,25(OH)_2_D-mediated rapid activation of voltage-dependent Ca channels exists in cardiac muscle cells [6]. Consequently, 1,25(OH)_2_D administration can normalize the impaired contractility of the myocardium that is observed under experimental vitamin D deficiency [7]. Renal synthesis of 1,25(OH)_2_D is suppressed by the phosphaturic hormone fibroblast growth factor-23 (FGF-23). FGF-23 is produced by bone cells, is stimulated by high serum phosphate levels, and promotes phosphaturia to maintain serum phosphate levels within the normal range [8].

In HF patients, low 1,25(OH)_2_D levels and high FGF-23 levels are independently associated with poor clinical outcome [8–12]. Patients with failing hearts have very low 1,25(OH)_2_D levels and extremely high FGF-23 levels [9]. In end-stage HF, left-ventricular assist device (LVAD) implants are increasingly used as a bridge to transplant or destination therapy [13,14]. Although LVAD implants increase survival significantly [13], mortality rates remain high in LVAD-supported HF patients [15].

Little is currently known about the effect of LVAD implants on calciotropic and phosphaturic hormones such as 1,25(OH)_2_D and FGF-23. The present study therefore aimed to investigate the postoperative time course of the aforementioned hormones in patients supported with an LVAD implant.

## Methods

### Patients

Sixty-nine patients of the Heart & Diabetes Center NRW, Germany, where preoperative and postoperative blood samples were available, were included in the present study. We used biobank plasma samples, stored at -80°C that were collected for the Mechanical Circulatory System Program at the time of VAD implantation, and at different time points thereafter. The aim of the biobank is to address novel research questions in the field of end-stage HF. Due to frequent hospitalization, patients who did not survive the first postoperative year were overrepresented in the present study cohort (45 non-survivors, 24 survivors). The calciotropic and phosphaturic hormones 25OHD, 1,25(OH)_2_D, PTH, and FGF-23 were analyzed in samples collected just prior to LVAD implantation (within the last 3 days before LVAD implantation; designated t0), and in samples collected 12 days postoperatively (IQR: 10–16 days), 30 days postoperatively (IQR: 24–35 days), 83 days postoperatively (IQR: 55–103 days), and 300 days postoperatively (IQR: 237–374 days) (designated t1, t2, t3, and t4, respectively). Only patients with HeartMate II and HeartWare LVAD implants were considered for the present study. The surgical technique of device implantation, as well as medication use during follow-up, is described elsewhere [15]. Written informed consent for biobanking of plasma samples was received from all patients. Moreover, study procedures were approved by the ethics committee of the Ruhr University Bochum at Bad Oeynhausen, Germany.

### Biochemical Analyses

Postoperative PTH, 25OHD, and FGF-23 levels were analyzed with the same test kits as the previously published preoperative data [15]. Briefly, c-terminal FGF-23 was measured using an ELISA test kit provided by Immutopics (San Clemente, CA). The reference range is < 100 RU/ml. 25OHD levels (sum of 25OHD_2_ and 25OHD_3_), which are the generally accepted indicator of vitamin D status, were analyzed by the autoanalyzer Liaison (DiaSorin,Stillwater, MN, USA). According to the Institute of Medicine [16], 25OHD levels < 12 ng/ml (multiply by 2.496 to convert into nmol/l) are classified as deficient, and values between 12 and 20 ng/ml as inadequate. The DiaSorin autoanayzer was also used instead of the previously used liquid chromatography tandem mass spectrometry method to measure preoperative and postoperative 1,25(OH)_2_D levels (sum of 1,25(OH)_2_D_2_ and 1,25(OH)_2_D_3_). This method requires only 75 μl of sample volume instead of the 500 μl needed by liquid chromatography tandem mass spectrometry method. The limit of 1,25(OH)_2_D quantitation is 5 pg/ml (multiply by 2.4 to convert into pmol/l), and we considered values below this limit as 4.5 pg/ml. The reference range is considered to be 20 to 79 pg/ml. Intact PTH was measured by an ELISA test kit provided by Biomerica, Irvine, CA, USA. Values between 10 and 60 pg/ml are usually classified as adequate. Creatinine levels were determined by standard procedure and estimated glomerular filtration rate (eGFR) was calculated by using the Modification of Diet in Renal Disease formula.

### Statistics

Categorical variables are reported using the number (n) and percentage of observations. Since calciotropic/phosphaturic hormones were non-normally distributed, as checked by the Kolmogorov Smirnoff test, these data are presented as median and interquartile range (IQR). To assess time effects on calciotropic/phosphaturic hormones, the Kruskal Wallis test was used. In case of significant time effects, post-hoc analyses using the Mann-Whitney test were performed to assess differences between specific time points. The Mann-Whitney test was also used to assess differences at specific time points between different subgroups. Spearman’s rank sum test was applied to assess interrelationships between eGFR, FGF-23, vitamin D metabolites, and PTH. Multiple regression analysis was used to assess independent predictors of circulating FGF-23 and 1,25(OH)_2_D levels. Non-normally distributed biochemical variables were log(e) transformed prior to these analyses to achieve a normal distribution. P-values <0.05 were considered statistically significant. We applied the statistical software package SPSS, version 21 (IBM Corp, Armonk, NY, USA) to perform the analyses.

## Results

The baseline and clinical characteristics of the patients are given in Table 1.

**Table 1:** Baseline characteristics of the patients at enrollment.

Parameter	All Cases (n = 69)	Survivors(n = 24)	Non-Survivors(n = 45)	P-value
Age, years	58 (48;65)	51.5 (45.3;57.0)	62 (53;67)	0.005
Male Gender, n (%)	62 (89.9)	21 (87.5)	41 (91.1)	0.687
Body Mass Index, kg/m^2^	24.7 (22.5;27.3)	24.1 (22.7;27.2)	25.2 (21.8;27.3)	0.940
Sort of Device				
HeartMate II	23 (33.3)	5 (20.8)	18 (40.0)	0.179
HeartWare	46 (66.7)	19 (79.2)	27 (60.0)	0.179
Diagnosis				
Dilated Cardiomyopathy	27 (39.1)	11 (45.8)	16 (35.6)	0.446
Ischemic Cardiomyopathy	38 (55.1)	11 (45.8)	27 (60.0)	0.314
Others	4 (5.8)	2 (8.3)	2 (4.4)	0.606
Hemodynamic Function				
LVEF, %	20 (15;26)	20 (16;25)	20 (15;26)	0.862
LVEDD, mm	70 (63;74)	71 (63;81)	68 (63;73)	0.388
Cardiac Index, L/min/m^2^	2.4 (1.8;2.7)	2.1 (1.6;2.6)	2.4 (1.8;2.8)	0.467
PAP, mmHg	31 (22;39)	34 (23;40)	28 (22;38)	0.208
PVR, dyn·s/cm^5^	188 (128;280)	253 (150;327)	159 (118;269)	0.053
PCWP, mmHg	19 (12;24)	22 (16;24)	18 (10;29)	0.659
INTERMACS Level				
1	5 (7.2)	0 (0)	5 (11.1)	0.155
2	15 (21.7)	9 (37.5)	6 (13.3)	0.031
3	30 (43.5)	10 (41.7))	20 (44.4)	>0.999
4	19 (27.5)	5 (20.8)	14 (31.1)	0.411
eGFR, ml/min/1.73m^2^	55.6 (39.4;81.5)	74.9 (49.7;95.6)	50.3 (34.6;70.1)	0.018
Hemoglobin (g/dl)	11.0 (10.1;12.8)	10.9 (9.6;12.0)	11.2(10.1;12.8)	0.508
White Blood Cell Counts, 10^9^/l	8.6 (7.0;10.9)	9.4 (7.8 (12.4)	8.0 (6.7;10.8)	0.147

Data are expressed as median and interquartile range or number (percentage). Abbreviations: eGFR, estimated glomerular filtration rate; LVEF, left ventricular ejection fraction; LVEDD, left ventricular enddiastolic diameter; PAP, pulmonary artery pressure; PVR, pulmonary vascular resistance; PCWP, pulmonary capillary wedge pressure

Most patients were male. One third of the study cohort had HeartMate II implants and two-thirds had HeartWare implants. The cause of HF was ischemic cardiomyopathy in the majority of patients (55.1%), dilated cardiomyopathy in 39.1% and other causes in only 5.8%. Of the 69 patients, 5, 15, 30, and 19 patients had INTERMACS (Interagency Registry for Mechanically Assisted Circulatory Support) level 1, 2, 3, and 4, respectively. Survivors and non-survivors did not differ significantly according to gender distribution, body mass index, diagnosis, hemodynamic function, INTERMACS levels, sort of device, hemoglobin levels, and white blood cell counts. However, non-survivors were significantly older and had lower baseline eGFR values than survivors.

In total, 264 blood samples (69 preoperative samples and 195 postoperative plasma samples) could be analyzed. At t0, FGF-23 levels were above its reference range of 100 RU/ml and 1,25(OH)_2_D levels were below its reference range of 20 pg/ml in 98.6% of patients (68 out of 69 patients) and 81.2% of patients (56 out of 69 patients), respectively. Moreover, elevated PTH and deficient 25OHD levels were present at t0 in 19 (27.3%) and 40 patients (58.2%), respectively. There were significant time effects on eGFR, FGF-23 and 1,25(OH)_2_D levels, but not on PTH or 25OHD levels (Table 2). Post-hoc analysis revealed a transient increase in eGFR, a transient decrease in FGF-23, and a continuous increase in 1,25(OH)_2_D values.

**Table 2:** Kidney function and calciotropic and phosphaturic hormones before and at different time points after left ventricular assist device implantation.

Parameter	t0	t1	t2	t3	t4	P-value
	n = 69	n = 51	n = 62	n = 47	n = 35	
eGFR (ml/min/1.73m^2^)	56 (39;82)	82 (54;122)**	86.5 (57;107)***	72 (41;100)	58 (35;81)	<0.001
25OHD (ng/ml)	11.8 (8.9;17.9)	11.3 (8.5;16.8)	10.9 (8.0;14.7)	11.1 (8.3;14.6)	11.1 (8.3;14.6)	0.564
PTH (pg/ml)	42 (32;95)	68 (37;107)	48 (24;77)	52 (37;84)	52 (37;83)	0.179
1,25(OH)_2_D (pg/ml)	7.7 (4.9;13.7)	7.8 (4.9;11.4)	8.3 (4.9;15.5)	12.1 (6.1;21.6)*	12.1 (6.1;21.6)*	0.024
FGF-23 (RU/ml)	683 (298;2356)	964 (521;2023)	417 (294;984)*	438 (281;1106)**	535 (334;1506)	0.008

Data are expressed as median and interquartile range. Abbreviations: eGFR, estimated glomerular filtration rate; 25OHD, 25-hydroxyvitamin D, PTH, parathyroid hormone; 1,25(OH)_2_D, 1,25-dihydroxyvitamin D; FGF-23, fibroblast growth factor-23; t0, before assist device implantation; t1,12 days post-intervention; t2, 30 days post-intervention; t3, 83 days post-intervention; t4, 300 days post-intervention *,**,*** significant different vs. t0, *P<0.05;**P<0.01;***P<0.001

Table 3 summarizes kidney function and hormone levels according to survival status: In non-survivors, FGF-23 and 1,25(OH)_2_D did not change significantly post-interventionally. In survivors, however, 1,25(OH)_2_D increased into the reference range. At t4, 1,25(OH)_2_D levels were significantly higher in survivors than in non-survivors (P = 0.006). In both, non-survivors and survivors, FGF-23 remained markedly elevated until t4. The FGF-23 levels did not differ between survivors and non-survivors at any time point. eGFR values were significantly higher in survivors than in non-survivors at baseline and t1 (P = 0.018 and 0.024), but not thereafter (P>0.05).

**Table 3:** Kidney function and calciotropic and phosphaturic hormones before and at different time points after left ventricular assist device implantation, broken down by survival status.

Parameter	t0	t1	t2	t3	t4	P-value
**Non-Survivors (n = 45)**	**n = 45**	**n = 32**	**n = 41**	**n = 32**	**n = 19**	
eGFR (ml/min/1.73m^2^)	50 (35;70)*	75 (41;107)**	84 (54;104)	71 (38;95)	50 (34;80)	0.013
25OHD (ng/ml)	10.8 (8.2;17.6)	11.0 (8.6;15.8)	11.0 (8.0;13.7)	10.3 (8.5;14.7)	11.1 (9.2;14.6)	0.988
PTH (pg/ml)	40 (32;95)	76 (37;129)	48 (22;73)	39 (20;62)	49 (21;65)	0.058
1,25(OH)_2_D (pg/ml)	7.7 (4.9;12.5)	7.3 (4.9;11.4)	7.5 (4.9;12.6)	10.3 (6.1;20.1)	10.6 (4.9;15.5)	0.599
FGF-23 (RU/ml)	612 (267;2356)	963 (381;2021)	409 (251;1007)*	435 (323;1217)	500 (334;1513)	0.173
**Survivors (n = 24)**	**n = 24**	**n = 19**	**n = 21**	**n = 15**	**n = 16**	
eGFR (ml/min/1.73m^2^)	75 (50;96)^+^	107 (68;138)*,^+^	95 (74;115)*	72 (58;111)	62 (49;84)	0.012
25OHD (ng/ml)	13.1 (11.1;21.5)	11.8 (7.7;18.5)	10.8 (7.6;16.0)	12.3 (7.1;13.5)	11.3 (6.5;14.3)	0.271
PTH (pg/ml)	49 (33;102)	54 (34;76)	46 (25;94)	73 (28;91)	64 (47;88)	0.696
1,25(OH)_2_D (pg/ml)	8.1 (4.9;20.0)	9.6 (4.9;11.5)	12.4 (4.9;18.1)	12.8 (8.8;34.7)	22.2 (7.6;28.0)*,^++^	0.016
FGF-23 (RU/ml)	1055 (340;3301)	1204 (545;2297)	425 (339;957)	477 (249;795)	539 (212;1096)	0.062

Data are expressed as median and interquartile range. Abbreviations: eGFR, estimated glomerular filtration rate; 25OHD, 25-hydroxyvitamin D, PTH, parathyroid hormone; 1,25(OH)_2_D, 1,25-dihydroxyvitamin D; FGF-23, fibroblast growth factor-23; t0, before assist device implantation; t1,12 days post-intervention; t2, 30 days post-intervention; t3, 83 days post-intervention; t4, 300 days post-intervention *,** significant different vs. t0, *P<0.05;**P<0.01; +,++ significant different vs. non-survivors at the same time point, +P<0.05; ++P<0.01

Based on the 264 plasma samples, Table 4 summarizes the relationships between the measured hormones, kidney function, body mass index, and age: Circulating FGF-23 was inversely related to eGFR and circulating 1,25(OH)_2_D, but was unrelated to PTH or 25OHD. Circulating 1,25(OH)_2_D was also positively correlated with 25OHD, and PTH, and was positively correlated with eGFR. Multiple regression analysis revealed that FGF-23, eGFR, 25OHD, and PTH were independently related to circulating 1,25(OH)_2_D levels (multiple R^2^ = 0.305;P<0.001), and that eGFR, age, 25OHD, and 1,25(OH)_2_D were independently related to circulating FGF-23 levels (multiple R^2^ = 0.239;P = 0.001).

**Table 4:** Interrelationships between study variables according to Spearman’s rank correlation coefficient (n = 264).

	**eGFR**	**25OHD**	**PTH**	**1,25(OH)**_**2**_**D**	**FGF-23**	**BMI**	**Age**
**eGFR**	-	0.016	-0.138*	0.271***	0.376***	0.215***	0.292***
**25OHD**	0.016	-	-0.158**	0.220***	0.003	-0.107	0.310***
**PTH**	-0.158**	-0.158**	-	0.269***	0.003	0.178**	0.024
**1,25(OH)**_**2**_**D**	0.220***	0.220***	0.269***	-	0.404***	-0.054	0.076
**FGF-23**	-0.376**	0.003	0.003	-0.404***	-	0.064	-0.035
**BMI**	-0.215**	-0.107	0.178**	-0.054	0.064	-	0.130*
**Age**	0.292***	0.310***	0.024	0.076	-0.035	0.130*	-

*,**,*** significant different vs. t0, *P<0.05;**P<0.01;***P<0.001

## Discussion

The present study indicates transient effects of LVAD implantation on kidney function and FGF-23 levels and a significant increase in circulating 1,25(OH)_2_D levels until the end of the follow-up period. These latter results were largely influenced by those patients who survived the first post-implant year, and were not seen in non-survivors.

To the best of our knowledge, this is the first publication to address the effect of LVAD implants on FGF-23 and 1,25(OH)_2_D levels. Our data demonstrate that end-stage heart failure patients have very high FGF-23 values and very low 1,25(OH)_2_D levels before and after LVAD implantation. We [9,11] and others [8,10,12] have already reported that elevated FGF-23 levels and low 1,25(OH)_2_D levels are both independently associated with poor clinical outcome in HF patients.

The transient increase in eGFR values after LVAD implantation is in agreement with earlier findings in end-stage heart failure patients with LVAD implants [17]. This transient effect on kidney function was observed in both survivors and non-survivors and may at least in part explain why FGF-23 levels did not continuously decline after LVAD implantation and remained markedly elevated until t4, because the synthesis of FGF-23 is increased by elevated serum phosphate levels [8], e.g. as a consequence of impaired kidney function [18]. However, an alternative explanation for the lack of a permanent suppression of FGF-23 levels by LVAD implants is also possible and may involve the altered energy and phosphate metabolism of the failing heart. The heart consumes more energy than any other organ and the failing heart is considered to be an engine out of fuel [19]. Even in HF patients without severe kidney disease serum phosphate levels are elevated [1]. Under conditions of stress, cardiomyocytes are able to synthesize FGF-23 by themselves [20]. Although patients with LVAD implants show some metabolic improvements in glucose metabolism and tissue inflammation [21,22], other derangements such as indices of myocardial fibrosis and impaired amino acid and creatine metabolism persist [21,23]. Therefore, the elevated FGF-23 levels after LVAD implantation may at least in part indicate a permanently impaired cardiac function.

In the present investigation, circulating 1,25(OH)_2_D levels improved slightly, but significantly. Nevertheless, values remained on average below the reference range of 1,25(OH)_2_D pg/ml. It is noteworthy that in our study well-known predictors of 1,25(OH)_2_D synthesis, such as 25OHD, kidney function, FGF-23, and PTH, could only partly explain the variations in circulating 1,25(OH)_2_D, and that 1,25(OH)_2_D improved significantly only in survivors, but not in non-survivors. Survivors were significantly younger and had higher baseline eGFR values than non-survivors. An earlier study in cardiac surgical patients indicated an independent inverse association between circulating 1,25(OH)_2_D levels and clinical outcome [24]. In contrast to the present study, levels of 1,25(OH)_2_D were also inversely related to age in that earlier study. The mechanisms being responsible for the increase in circulating 1,25(OH)_2_D in the survivors of the present study remain unclear. Notably, circulating 1,25(OH)_2_D levels are also significantly and inversely related to the inflammation marker CRP [25]. Since LVAD implantation seems to reduce inflammatory processes [22], the slight improvement in circulating 1,25(OH)_2_D may be the result of some metabolic improvements, at least in survivors. The present findings also confirm earlier results of an inverse association between postoperative 1,25(OH)_2_D levels and 1-year mortality in heart transplant recipients [26]. In that earlier study, mortality was highest at 1,25(OH)_2_D levels < 11 pg/ml (32.1%), intermediate at levels between 11 and 18 pg/ml (13.2%) and lowest at levels > 18 pg/ml (3.7%). Similar to circulating 1,25(OH)_2_D, there are also time effects of LVAD implants on natriuretic peptides in end-stage HF patients [27], and these peptides can also provide information for identifying patients who are more likely to recover.

As mentioned before, 1,25(OH)_2_D plays a pivotal role in the intracellular handling of ionized calcium [5,6] and 1,25(OH)_2_D administration can normalize the impaired contractility of the myocardium that is observed under experimental vitamin D deficiency [7]. Although cardiomyocytes possess 1-α-hydroxylase activity [28], the heart muscle probably depends at least in part on circulating 1,25(OH)_2_D levels. This assumption is supported by experimental data demonstrating that overall deletion and cardiomyocyte-specific deletion of the vitamin D receptor results in cardiac hypertrophy [29,30], whereas treatment of neonatal cardiomyocytes with 1,25(OH)_2_D can partially suppress cardiac hypertrophy [30].

Similar to the present study, earlier studies have reported deficient circulating 25OHD levels, e.g. levels < 12 ng/ml, in HF patients [1]. Moreover, significantly lower 1,25(OH)_2_D levels were present in HF patients than in (elderly) control patients, and lowest 1,25(OH)_2_D levels were observed in those patients with early onset of the disease [1]. The assumption that low levels of 1,25(OH)_2_D contribute to poor clinical outcome in HF is in agreement with two meta-analyses of randomized controlled trials: Vitamin D supplements improve clinical outcome in HF patients [31], and increase circulating 1,25(OH)_2_D levels on average by 7.8 pg/ml (95% CI, 3.8–11.8 pg/ml) [32]. However, the present findings also illustrate that 1,25(OH)_2_D regulation is complex. Therefore, we should not be too enthusiastic to believe that simple vitamin D supplementation would be able to restore all vitamin D-related derangements in end-stage HF.

Our study has both strengths and limitations. The strengths include the prospective study design, the multiple blood drawings at different time points, the measurement of various hormones of calcium and phosphate metabolism, and the relatively large cohort of patients with LVAD implants. One limitation is that even in survivors blood samples were not available for all patients at all time points. A second limitation is the lack of a healthy control group or a group with other cardiovascular diseases. A third limitation is that no biomarkers of heart failure such as brain natriuretic peptide or N-terminal pro-atrial natriuretic peptide were available to assess the association between the severity of heart failure with the concentrations of the measured hormones of calcium and phosphate metabolism. Moreover, no postoperative data on hemodynamic parameters were available for this study.

In conclusion, LVAD implantation has only moderate effects on calciotropic and phosphaturic hormones such as 1,25(OH)_2_D and FGF-23. Results are in general agreement with the hypothesis that 1,25(OH)_2_D, and probably also FGF-23, plays a pivotal role in the pathogenesis of HF and that mortality remain high in end-stage HF patients, despite LVAD implantation. Future studies are necessary to investigate in more detail the associations of disease severity, LVAD implantation, calciotropic and phosphaturic hormones, and clinical outcomes.
